# Topics of Public Interest

**Published:** 1905-08

**Authors:** 


### TOPICS OF PUBLIC INTEREST.

### President Roosevelt's Address to the Associated Physicians of Long Island.

THE associated physicians of Long Island held their annual meeting at Oyster Bay, July 12, 1905, and were addressed by the President of the United States.

The President rode down from Sagamore Hill, wearing his broad brimmed straw hat and his white flannels, to address the doctors, 300 strong, in the high school building. It was about 3.10 o'clock when his team turned from the Cove road up Anstey street, and it was rather fortunate that he was a little tardy, for the physicians were late in reaching town. The train bringing them to Oyster Bay fell in behind a stalled circus train somewhere up the road, so it was nearly 3 o'clock when they reached the village. Only by making a perspiring sprint up the hill to the schoolhouse were they able to reach the hall and find their seats before the President arrived.

The President was introduced to the officers of the physicians' association on the lawn in front of the school. When he reached the stage inside the building he was presented to the assemblage by Dr. W. B. Savage, of Islip, the president of the association. President Roosevelt spoke as follows:

*Mr. President, Members of the Association, Friends and Neighbors.*—I needed no invitation to come before you today. All I needed was permission. As soon as I learned that this association was to meet in our village I felt that I must take advantage of the opportunity to say a word of greeting to you in person.

Of course it is almost needless to say that there is not and cannot be any other lay profession the members of which occupy such a dual position, each side of which is of such importance, for the doctor has on the one hand to be the most thoroughly educated man in applied science that there is in the country, and, on the other hand, as every layman knows, and doubtless many a layman in the circle of acquaintance of each of you would gladly testify, the doctor gradually becomes the closest friend to more different people than would be possible in any other profession. The feeling that a man has toward the one human being to whom he turns either in time of sickness for himself or, what is far more important, in the time of sickness of those closest and dearest to him, cannot but be of a peculiar kind. He cannot but have a feeling for him such as he has for no other man. The doctor must, therefore, to the greatest degree, develop both sides of his nature, develop his nature along the two sides of his duties, although in the case of any other man you would call him a mighty good citizen if he developed only on one side. The scientific man who is really a first class scientific man has a claim upon the gratitude of all the country. The man who is a first class neighbor and is always called in in time of trouble by his neighbor has an equal claim upon society at large. But the doctor has both claims. Yet, in addition to filling both of these functions he may fill many other functions. He may have served in the civil war, he may have rendered the greatest possible service to the community along a dozen different lines.

### SANITARY WORK IN PANAMA.

Take, for instance, just what is being done in one of the great works of this country at the present time—digging the Panama Canal. That is a work that only a big nation could undertake or that a big nation could do, and it is a work for all mankind. And the condition precedent upon success in that work is having the proper type of medical work as a preliminary. That is the first condition upon the meeting of which much depends our success in solving the engineering and administrative problems of the work itself. I am happy to say that the work is being admirably done, and I am particularly glad to have this chance of saying it.

Now and then some alarmist report will come from Panama. Just a couple of weeks ago there seemed to be a succession of people coming up from Panama, each one of whom had some tale or other to tell. You will always find in any battle, even if it is a victorious battle, that in the rear you meet a number of gentlemen who are glad that they are not at the front; who, if they have unfortunately got at the front, have come away, and who justify their absence from the front by telling tales of how everything has gone wrong there. Now, the people who flee from Panama will carry up here just such stories as the people who flee from the forefront of a battle carry to the rear with them. The people to whom this country owes, and will owe, so much are these who stay down there and do not talk, but do their work, and do it well.

Now, of course, in doing a great work like that in the tropics, in a region which, until this government took hold of it, was accounted to be a region exceptionally unhealthy, we are going to have trouble, have some yellow fever, have a good deal of malarial fever, and suffer more from the latter than from the yellow fever, although we will hear nothing like the talk about it. We will have every now and then troubles as regards hygiene, just as we will have trouble in the engineering problems, just as occasionally we will have trouble in the administrative work. Whenever one of those troubles comes there will be a large number of excellent but timid men who will at once say what an awful calamity it is, and express the deepest sorrow and concern and be rather inclined to the belief that the whole thing is a failure. It will not be a failure. It will be a success; and it will be a success because we shall treat every little check, not as a reason for abandoning the work, but as a reason for altering and bettering our plans so as to make it impossible that that particular check shall happen again.

LEONARD WOOD PRAISED.

What is being done in Panama is but a sample of the things that this country has done during the last few years, of the things in which your profession has had so prominent a part. Take what we did in Cuba, where we tried the experiment, which had not been tried for four hundred years, of cleaning the cities. One of the most important items of the work done by our government in Cuba was the work of hygiene, the work of cleaning and disinfecting the cities so as to minimise the chance for yellow fever, so as to do away with as many as possible of the conditions that told for disease. This country has never done better work, that is, work that reflected more honor upon the country, or for humanity at large, than the work done in Cuba. And the man who above all others will be responsible for doing that work so well was a member of your profession, who, when the call to arms came, himself went as a soldier to the field, the present Major General Leonard Wood. Leonard Wood did in Cuba just the kind of work that, for instance, Lord Cromer has done in Egypt. We have not been able to reward Wood in anything like the proportion that services such as his would have been rewarded in any other country of the first rank in the world; and there have been no meaner and more unpleasant manifestations in all our public history than the feelings of envy and jealousy manifested toward Wood. And the foul assaults and attacks made upon him, gentlemen, were largely because they grudged the fact that this admirable military officer should have been a doctor.

After the President had retired from the hall, the association unanimously elected him and General Leonard Wood honorary members.—*The Tribune.*

---

### State Education Department to Chancellor Reid.

DR. A. S. DRAPER, commissioner of education, his assistants and the chiefs of divisions in the state department of education sent to Whitelaw Reid, chancellor of the university of the State of New York, a congratulatory letter on the eve of his departure for his post as ambassador to the court of Saint James's. A messenger from the department was sent to New York and presented the letter to Mr. Reid the evening before he sailed for England. The letter of Commissioner Draper and his associates is as follows:

University of the State of New York,
Education Department,
Albany, May 26, 1905.

The Honorable Whitelaw Reid, LL.D., Chancellor of the University of the State of New York and Ambassador of the United States to Great Britain:

*Sir*—Upon the occasion of your departure from the country in pursuance of the highly honorable mission with which you have been charged by the president, the commissioner of education and the assistant commissioners, the secretary to the commissioner and the heads of divisions of the education department, desire to express their cordial appreciation of the propriety of your appointment as ambassador to the court of Saint James's. Your attainments in letters and in statesmanship, your distinguished public services at home, your ample diplomatic experience and the uniform courtesy of your deportment combine in the assurance that the republic will be eminently well represented in its most important and dignified diplomatic station. Not only will an illustrious succession be maintained in your person, but, through your offices, the ties of amity now happily existing between the two great Anglo-Saxon nations will be preserved and strengthened.

And yet it must be admitted that our gratulation is tempered by the regret that your sojourn abroad will temporarily sever your intimate relations with the university and with the activities of American education. For twenty-seven years you have been conspicuously and persuasively identified with the conduct of the university. Its usefulness and the expansion of its activities during that period are, in considerable measure, due to your counsel and direction. Your selection as chancellor, under the act unifying the administration of all of the educational institutions and activities of the state of New York, was the just recognition of your long and arduous labors for the promotion of higher learning, and the conviction of your associates that they could not more clearly define the purpose and scope of the new administration than in your preferment—a conviction in which it may be permitted the servants of that administration cordially to concur. If, for a time, the university must lose the benefit of your immediate oversight and advice, it will retain your constant concern and the valuable ministrations you may render it even in your absence.

It may not be too much to expect that your advantageous position abroad will enable you to communicate to us much concerning educational progress in Europe which will be of decisive aid to us in the administration and the energising of our own work.

With earnest wishes for a prosperous voyage, a renowned fulfilment of your exalted trust and a return in due season to this commonwealth and to the position in the government of the university which you have already signally adorned, we are, respectfully and sincerely yours,

Andrew S. Draper,

Commissioner of Education.

Howard J. Rogers,

First Asst. Commissioner.

Edward J. Goodwin,

Second Asst. Commissioner.

Augustus S. Downing,

Third Asst. Commissioner.

H. H. Horner,

Sec'y to the Commissioner.

Melvil Dewey,

Director of Libraries Div'n.

John M. Clarke,

Director of Science Div'n.

William Mason,

Chief, Accounts Div'n.

James D. Sullivan,

Chief, Attendance Div'n.

Charles F. Wheelock,

Chief, Examinations Div'n.

Frank H. Wood,

Chief, Inspections Div'n.

Thomas E. Finegan,

Chief, Law Div'n.

Charles E. Fitch,

Chief, Records Div'n.

Hiram C. Case,

Chief, Statistics Div'n.

---

### The American Medical Association.

WHAT IT STANDS FOR—RESEARCH AND PROGRESS ARE ITS AIMS AND THOUSANDS OF PHYSICIANS THROUGHOUT COUNTRY BENEFIT BY ITS ACCOMPLISHMENTS.

Dr. L. S. McMurtry, of Louisville, the reigning president, was interviewed by a member of the staff of the *Morning Oregonian* at Portland, soon after his arrival, and that famous newspaper published the conversation in its issue of July 10, 1905. The story of the newspaper man runs as follows:

"The American Medical Association is a post-graduate college at which men of national attainment gather annually to exchange ideas and to discuss the results and findings of their year's work and investigations. It has no politics and many of the members will return to their homes after the sessions without knowing who has been elected the president of the association. Its object is to promote the science and art of medicine, to unite in one compact organisation the medical profession of the United States and to elevate its standards."

Dr. McMurtry, being president of the American Medical Association and therefore the dean of this great national post-graduate college of eminent physicians and surgeons, sat last night in his room at the Portland Hotel and told of the aims and objects of the organisation. After long search the man who will wield the gavel during the convention to be held in the city this week, was found in one corner of the lobby surrounded by members of the association interested in the coming meetings.

TELLS OF THE PROFESSION.

"Come upstairs," he said when told he was about to be interviewed. "We can talk better there." He led the way around innumerable turns in the corridor and opened the door.

"Now," he said, "if you know how to turn on those lights I will let you do it. I have not been able to find any button to push."

The lights on, President McMurtry settled in his chair and told of the history of the organisation and what it stood for and had accomplished.

"The American Medical Association," said the speaker, relapsing into history, "was founded in 1846 and is the largest medical association in the world. Last year it met at Atlantic City and the year before that at New Orleans. Since its membership is made up from the progressive medical men throughout the United States the meeting places are chosen in different sections of the country in order to give the members in each district an opportunity to attend, while at the same time the members are given the privilege of visiting the different sections of the country.

"The object of the association is to promote the science and art of medicine, to unite the medical profession into one compact organisation and to elevate the standards of the profession. It is an association to the sessions of which the medical men of the nation may come and learn of the advance made in the year through the researches and investigations of the men who have attained high place in the ranks of the profession, men of national and oftentimes of worldwide reputation.

ITS SCIENTIFIC WORK.

"Its scientific work is carried on in 12 sections representing the various specialties and departments of procedure. In these different sections the men engaged in scientific research present the results of their investigations by written essays or papers which are discussed by those attending the congress.

"The medical profession is the most progressive of all for the reason that medicine is not yet ranked among the exact sciences. At these meetings the methods of treatment and operation and of procedure are discussed and presented to men engaged in the same lines of investigation and practice.

"The association embraces in its membership all of the progressive element of the profession in the United States and is in effect a great post-graduate college in which the men of attainment in the profession meet to exchange ideas and results.

DIFFUSION OF RESEARCH.

"For the diffusion of its researches the association publishes a weekly journal which has come to be the foremost medical publication of the world. The association owns valuable property in Chicago where the journal is published, together with other medical publications. The journal has a weekly circulation of 35,000 copies, the subscriptions being distributed throughout the United States."

Turning from history Dr. McMurtry chatted pleasantly of the work done by the association and of the difference between the physician of the pioneer days and of the present time. The association, the speaker held, was responsible in large part for the advancement made by the medical profession of the union. It kept alive and fostered the spirit of progress more than any one agency, as it brought every man of progressive ideas close in touch and harmony with his fellows.

OLD METHODS GONE.

"The old-time man who studied a few short months in some office and then went forth to cure all ills had passed and in his place had come the specialist, the man highly trained to do some certain branch well, the man who knew his subject as thoroughly as it might be given in the power of man to understand. Thus it was that the association had done and would continue to do much excellent work for the advancement of the profession. The association was a college from which none could ever graduate, for it would ever hold new things to be learned and taught, new investigations and discoveries to be made, new results to be achieved."

---

Two discoveries are imputed to Dr. Koch by cable dispatches as the result of his recent visit to East Africa. One—that the microbe of sleeping sickness exists in the tsetse fly—is not altogether original. By this he has merely confirmed the conclusions of Castellani, Sampon, and Brumpt, who made the same observations in 1902 and 1903. The other is apparently more novel. It is alleged that Dr. Koch has found a simple way to render the tsetse fly innocuous. If so, he has performed an excellent service. That insect is the carrier of the germs of several diseases. When it is itself infected with a certain kind, its bite is followed by an almost invariably fatal malady in horses and cattle, and extensive losses have been caused thereby in Africa. The pest seems to be unknown elsewhere. The sleeping sickness is a human disorder, but it is a less serious evil.

---

M. Combes, recently premier of France, who is a physician, has returned to the practice of medicine in his native village.

